# Feature Statistics Modulate the Activation of Meaning During Spoken Word Processing

**DOI:** 10.1111/cogs.12234

**Published:** 2015-06-04

**Authors:** Barry J. Devereux, Kirsten I. Taylor, Billi Randall, Jeroen Geertzen, Lorraine K. Tyler

**Affiliations:** ^1^ Department of PsychologyCentre for Speech, Language and the BrainUniversity of Cambridge; ^2^ Department of GeriatricsMemory ClinicUniversity Hospital Basel

**Keywords:** Concepts, Semantic features, Spoken word processing, Lexical decision, Connectionist modeling, Attractor networks, Lexical semantics, Conceptual structure

## Abstract

Understanding spoken words involves a rapid mapping from speech to conceptual representations. One distributed feature‐based conceptual account assumes that the statistical characteristics of concepts’ features—the number of concepts they occur in (*distinctiveness*/*sharedness*) and likelihood of co‐occurrence (*correlational strength*)—determine conceptual activation. To test these claims, we investigated the role of distinctiveness/sharedness and correlational strength in speech‐to‐meaning mapping, using a lexical decision task and computational simulations. Responses were faster for concepts with higher sharedness, suggesting that shared features are facilitatory in tasks like lexical decision that require access to them. Correlational strength facilitated responses for slower participants, suggesting a time‐sensitive co‐occurrence‐driven settling mechanism. The computational simulation showed similar effects, with early effects of shared features and later effects of correlational strength. These results support a general‐to‐specific account of conceptual processing, whereby early activation of shared features is followed by the gradual emergence of a specific target representation.

## 1 Introduction

Understanding spoken words involves accessing and processing their conceptual representations. This requires the rapid and efficient mapping from speech input to word meanings, a process known to be influenced by a variety of lexical, phonological, semantic, and contextual factors (e.g., Grainger & Jacobs, 1996; Grondin, Lupker, & McRae, 2009; Hargreaves & Pexman, 2014; Marslen‐Wilson, 1987, 1990; Marslen‐Wilson & Tyler, 1980; McClelland & Elman, 1986; Moss, Ostrin, Tyler, & Marslen‐Wilson, 1995; Yap, Pexman, Wellsby, Hargreaves, & Huff, 2012). One approach to the representation of word meanings is framed in terms of distributed feature networks in which concepts correspond to patterns of activation across semantic feature units, such as *has eyes*,* has ears*, and *has stripes* (Cree, McNorgan, & McRae, 2006; Durrant‐Peatfield, Tyler, Moss, & Levy, 1997; Farah & McClelland, 1991; Greer et al., 2001; McRae, de Sa, & Seidenberg, 1997; Pexman, Lupker, & Hino, 2002; Tyler, Moss, Durrant‐Peatfield, & Levy, 2000). Within this framework, the statistical characteristics of features are claimed to determine how concepts are represented and processed during on‐line comprehension. Two of the key statistical characteristics that have been proposed are *correlational strength* and *distinctiveness/sharedness* (Clarke, Taylor, & Tyler, 2011; Cree et al., 2006; McRae, Cree, Seidenberg, & McNorgan, 2005; McRae et al., 1997; Moss, Tyler, & Taylor, 2007; Taylor, Devereux, Acres, Randall, & Tyler, 2012; Taylor, Moss, & Tyler, 2007; Tyler et al., 2013). Correlational strength is a measure of the tendency of one feature to co‐occur with other features; for example, *has eyes* and *has ears* co‐occur in the same concepts more often than *is grey* and *has teeth* (Durrant‐Peatfield et al., 1997; Keil, 1986; Malt & Smith, 1984; McRae et al., 1997; Rosch, Mervis, Gray, Johnson, & Boyes‐Braem, 1976). Most distributed, feature‐based models (e.g., Cree, McRae, & McNorgan, 1999; McRae et al., 1997; Randall, Moss, Rodd, Greer, & Tyler, 2004; Taylor et al., 2012) claim that the activation of features in the network leads to the coactivation of other features with which they are strongly correlated, or “connected.” Distributed models therefore propose a facilitatory role for correlational strength in the activation of conceptual representations, with weakly correlated features activating more slowly than strongly correlated features (Cree et al., 1999; McRae, Cree, Westmacott, & de Sa, 1999; McRae et al., 1997; Randall et al., 2004; Taylor, Salamoura, Randall, Moss, & Tyler, 2008; Taylor et al., 2012).

The second key feature statistic, distinctiveness/sharedness, is a continuous variable that varies between highly distinctive features that occur in few concepts (e.g., *has an udder*,* has a mane*) and highly shared features that occur in many concepts (e.g., *has a tail*,* has legs*). Some findings have suggested that distinctive features play a privileged role in the organization and activation of semantic knowledge—activating earlier and stronger than shared features during the computation of word meaning (e.g., Cree et al., 2006)—whereas other accounts have focused more on the kind of information represented in distinctive features, positing that the importance of distinctive and shared information differs as a function of information demands of the given task context (Bright, Moss, Longe, Stamatakis, & Tyler, 2007; Grondin et al., 2009; Taylor, Devereux, & Tyler, 2011; Taylor et al., 2012). For example, the conceptual structure account (CSA; Moss et al., 2007; Taylor et al., 2012, 2007; Tyler & Moss, 2001a; Tyler, Moss, et al., 2000) claims that highly distinctive features are required for basic‐level concept identification because they differentiate the target from similar concepts (e.g., the distinctive feature *has a mane* helps differentiate a lion from a tiger). Highly shared features (e.g., *has legs*) provide information about the semantic category or domain to which a concept belongs (e.g., *animal* or *living thing*) and so are sufficient for tasks which require broad category‐level differentiation rather than differentiation between similar concepts (Humphreys, Price, & Riddoch, 1999; Humphreys, Riddoch, & Quinlan, 1988; Lloyd‐Jones & Humphreys, 1997; Moss & Tyler, 1997; Moss, Tyler, Durrant‐Peatfield, & Bunn, 1998; Taylor et al., 2012, 2007).

An additional critical dimension in conceptual processing is the time course of activation of shared and distinctive properties. Our recent empirical work testing the CSA supports a model of semantic processing where the emergence of a conceptual representation evolves in a general‐to‐specific manner, with more general features (i.e., those shared by many different concepts) activating earlier, on average, than the more specific, distinctive information that distinguishes between similar concepts (Clarke, Devereux, Randall, & Tyler, in press; Clarke, Taylor, Devereux, Randall, & Tyler, 2013; Clarke et al., 2011; Taylor et al., 2011). A recent MEG experiment investigating the spatio‐temporal nature of object processing (Clarke et al., 2013) provides neurocognitive support for this general‐to‐specific hypothesis. In this study, participants identified visual objects for which feature statistic information was available. The influence of non‐semantic low‐level variables was controlled (just as was the case for words in the current study; see below), as was the proportion of visual semantic features for the concept. The MEG analyses showed that initial perceptual effects in the visual cortices were rapidly followed (within 120 ms) by effects of shared features in the ventral temporal lobe. Later (post‐200 ms), the effects of both shared and distinctive features were seen in the ventral temporal cortex. These results suggest that coarse, category‐level information which relies on the activation of shared features is available early in processing with distinctive information specific to the concept becoming available later. This general‐to‐specific model is similar to the notion of coarse‐to‐fine‐grained visual processing, where low spatial frequency information (such as the general shape of an object) is processed before more specific visual detail (Bar et al., 2006; Hegdé, 2008; Schendan & Stern, 2008).

**Figure 1:**
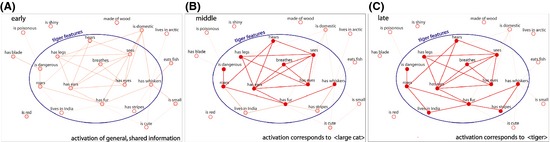
Cartoon illustrating the hypothesized effects of conceptual structure on the activation of the concept *tiger* over time. Small circles represent semantic features, and their color saturation represents the level of activation. Lines represent activation flow between features. (A) In the initial stages of processing, activation is spread diffusely over features throughout the semantic network. Shared features are initially weakly activated via the word form level, and many of these belong in the target representation (e.g., *has whiskers*) but others do not (e.g., *is domestic*). (B) Over time, mutually reinforcing activation gradually builds up between the correlated features within the concept, increasing overall activation within the target representation. The speed of this within‐concept coactivation depends on the mean correlational strength between features within the concept. In the context of a lexical decision task, within‐concept coactivation in stages (A) and (B) provides semantic evidence that the word denotes a real concept. (C) Weakly correlated distinctive features (*lives in India* and *has stripes* in this illustration) activate slower, facilitating tasks that require access to a specific representation, such as object naming (Taylor et al., 2012).

The general‐to‐specific hypothesis within the context of the CSA suggests that the earliest stages of conceptual processing should be primarily driven by the initial activation of many shared features throughout the semantic network (some of these features may be within the target concept, but many will be outside of it; Fig. 1A). Over time, as processing continues toward a specific target representation, the settling of activation will be driven by the mutual coactivation of features *within* the concept: Concepts with features that are strongly interconnected with each other will settle toward their target representation faster than concepts that have relatively weakly interconnected features (Cree et al., 1999; McRae et al., 1997, 1999). Thus, the influence of the correlational strength of a concept's features is predicted to build up over time and primarily affect later stages of processing, as correlated features within the target concept's representation become mutually coactivated (Fig. 1B; for a computational simulation on how feature correlation drives network settling over time, see Cree et al., 1999). The latest stages of processing reflect the activation of a specific target conceptual representation, that is, when distinctive features become fully integrated into the representation (Fig. 1C). Depending on the demands of the task, the earlier more general level of representation may be sufficient to perform the task and activation of distinctive concept‐specific information may not be required. The general‐to‐specific account incorporates the differing claims that have been made for distinctiveness/sharedness in conceptual processing by incorporating the dimension of time: Shared features may be important for early, more general activation, such as that required for category‐level judgements, whereas distinctive features become important at later stages of processing where a specific conceptual representation needs to be activated.

Could a similar general‐to‐specific mechanism underlie spoken word comprehension? According to interactive models of spoken word recognition such as the cohort model (Marslen‐Wilson, 1987), semantic activation begins relatively early during the time course of processing, before the point at which the word can be uniquely identified. Early semantic activation must therefore momentarily be driven by all words in the cohort of words that are consistent with the currently available speech input (e.g., on hearing the onset [bi:], the semantics of “beaker,” “beetle,” “beach,” etc., will recieve transient activation; Marslen‐Wilson, 1987, 1993; Zwitserlood, 1989). In a distributed, feature‐based framework, this early semantic activation must necessarily be partial and imprecise, consisting of an overlapping “blend” of constituent features from the cohort concepts (Gaskell & Marslen‐Wilson, 1997, 1999, 2002). An interesting question is whether some kinds of features are privileged over others within this initial activation blend; for example, features that are more central to the cohort concepts’ meaning may activate more strongly than features that are more peripheral to core meaning (Moss, McCormick, & Tyler, 1997). Thus, one possibility, consistent with the general‐to‐specific hypothesis, is that feature statistics may influence how strongly features within the blend are activated—shared features will on average be true of more cohort members and therefore may be more strongly activated. As the cohort gradually decreases in size, semantic activation becomes more specific, and distinctive information becomes relatively more activated.

In the present study, we used a lexical decision task to examine the role of feature statistics at different phases of the online processing of spoken words. The activation of semantic information is not logically required to decide whether an isolated word form is a real word (Grainger & Jacobs, 1996; Seidenberg & McClelland, 1989), but a large body of evidence attests to the influence of semantic information on lexical decisions (e.g., Balota, Ferraro, & Connor, 1991; Binder et al., 2003; Chumbley & Balota, 1984; Grondin et al., 2009; Hargreaves & Pexman, 2014; Hino & Lupker, 1996; James, 1975; Moss et al., 1995; Pexman, Hargreaves, Siakaluk, Bodner, & Pope, 2008; Pexman et al., 2002; Tyler, Voice, & Moss, 2000; Yap et al., 2012). Although non‐semantic factors may also be expected to influence decision latencies, the nature of the semantic effects on lexical decision performance is the focus of the present manuscript. The lexical decision task allows us to investigate our general‐to‐specific hypothesis, because the influence of feature sharedness/distinctiveness and correlational strength within the concept should vary at different stages of processing. In particular, the sharedness of a concept's features should determine the amount of initial activation in the semantic network. An alternative hypothesis can be generated from the findings of Cree et al. (2006). In a pair of feature verification tasks (in which (a) nouns were followed by features, and (b) features were followed by nouns), Cree et al. found that distinctive features were verified true of the noun more quickly than shared features. Cree et al. argued that distinctive features have a privileged status in the computation of word meaning and thus increased distinctiveness of a concept's features should facilitate processing (Cree et al., 2006; but see also Grondin et al., 2009).

We measured the influence of conceptual structure statistics on the on‐line processing of individual concepts, using omnibus variables that measure the overall distinctiveness/sharedness and overall correlational strength of features within the concept (see also Grondin et al., 2009; Taylor et al., 2012). Given that lexical decision latencies to spoken words are known to depend on a large number of lexical, phonological, and other conceptual variables, we used a regression design, enabling us to take the influence of these variables into account. We planned to measure the initial (compared to late) quality of information available by comparing fast as opposed to slow responders. Participants who respond more rapidly in the lexical decision task because of the influence of early general activation should show greater sensitivity to the overall sharedness of concepts’ features, with concepts that have many shared features, generating large amounts of activation over semantic space, being responded to faster than concepts with fewer shared features. However, slower participants may take longer to accrue sufficient semantic evidence before responding. Slower participants’ lexical decisions would then be more strongly influenced by the activation of a network of correlated features within the concept. For example, in deciding that *tiger* is a real word, slower participants may be more influenced by activation of the network of features corresponding to the category *large cat* because a coherent category‐level representation such as this is stronger evidence of lexical meaning (we therefore hypothesize that participants may respond around the stage depicted in Fig. 1B). In this scenario, the gradual mutual coactivation of interconnected features within the concept enables a positive decision, and the speed at which features of the concept coactivate, as measured by the mean correlational strength of shared features within the concept, has a stronger influence on decision latencies.

In a second experiment, we investigate the mechanics of the general‐to‐specific account and the role of feature statistics in semantic processing using a connectionist simulation of the mapping from spoken input to distributed meaning representations. We implemented an attractor model, based on the Cree et al. (2006) architecture and adapted to simulate the activation of word meaning from continuous speech input. The model allows us to examine how the activation levels of different kinds of features within target concepts evolve over time, through recurrent connectivity. In particular, we predicted that concepts with many highly shared features (i.e., low mean distinctiveness) would show stronger overall activation at earlier stages of word processing, compared with concepts with more distinctive features, for which activation levels would take longer to build up over time. Furthermore, we predicted that mean correlational strength of shared features in the target concepts would become gradually more facilitatory as activation settled toward the target representation.

In summary, the two experiments were designed to test predictions for the influence of feature statistics at different phases of the online processing of spoken words. For lexical decision, we predicted (a) a facilitatory effect of the degree of initial, general semantic activation (i.e., shared features), since lexical decision does not require basic‐level identification, and (b) a facilitatory effect of the degree of correlational strength between the features within the concept, particularly for slower participants who are more likely to base their responses on additional, later semantic information that yields a coherent category‐level representation. For the simulations of Experiment 2, we predicted (a) concepts with many highly shared features would generate higher levels of overall activation at early processing stages compared with concepts with more distinctive features, and (b) stronger overall activation for concepts with strong connections between features at later stages of processing.

## 2 Experiment 1: Behavioral study

### 2.1 Method

#### 2.1.1 Participants

Thirty‐four native British English speakers with normal hearing (aged 18–35) participated in the experiment. They were paid £6 for their participation.

#### 2.1.2 Design and materials

Stimuli were concepts from the McRae et al. (2005) property norms, modified for use with British English‐speaking participants (see Taylor et al., 2012, for details). Homophones (e.g., *bat*,* cap*, and *inn*) were excluded, leaving a total of 447 experimental items. Four hundred forty‐seven pronounceable non‐word stimuli were created by rotating the syllables from the real words. The words and non‐words were digitally recorded in a random order in a sound‐proof booth by a female native British English speaker.

We obtained the following information for each word: the number of phonemes, its log lemma frequency (from CELEX; Baayen, Pipenbrook, & Gulikers, 1995), stimulus duration in milliseconds, and familiarity (taken from the MRC Psycholinguistic Database (Coltheart, 1981) when available [68% of concepts] and otherwise from laboratory pretests [at least 15 participants per concept]). In order to avoid the high collinearity between number of phonemes and stimulus duration (*r *=* *.74), we reexpressed our phonological measure as a measure of the rate of phonemic change in the word (“phoneme rate,” i.e., number of phonemes divided by stimulus duration). This provided a conceptually easily interpretable measure of phonology that was relatively uncorrelated with duration (*r *=* *.17), and we predicted that the phonologically richer input of high phoneme rate items would be facilitatory (see, e.g., Baayen, Davidson, & Bates, 2008; Baayen, Feldman, & Schreuder, 2006; for similar reexpressions of collinear predictors). The number of features (NOF) associated with each concept, calculated from the anglicized McRae norms, was also included as a control variable. Concepts with more features will tend to generate richer semantic activation (all else being equal), and so we expect a facilitatory role for NOF (Pexman, Holyk, & Monfils, 2003; Pexman et al., 2002).

Two feature statistics, capturing different structural characteristics of each concept, were calculated. The *distinctiveness* of a feature was defined as the multiplicative inverse of the number of concepts that it occurs in (McRae et al., 2005; for example, feature occurring in two concepts has a distinctiveness of 0.5). Distinctiveness values therefore vary along a continuum from maximal distinctiveness (i.e., very high distinctiveness values) to maximal sharedness (i.e., very low distinctiveness values). Like McRae et al. (2005), we define a feature as *distinguishing* if it occurs in only one or two concepts and *shared* if it occurs in more than two concepts. For each concept, we calculate the *mean distinctiveness* of the concept's features (i.e., the average of the distinctiveness values for each feature in the concept).

The strength of correlation between a pair of features was calculated as the Pearson correlation between the production frequency vectors for the two features (McRae et al., 1997, 2005; Randall et al., 2004; Taylor et al., 2012). For each concept, we calculated the *mean correlational strength* between all pairs of significantly correlated features within the concept (Randall et al., 2004; Taylor et al., 2008, 2012). Since it has been argued that correlations with distinguishing features may be spurious (Cree et al., 2006), we calculated mean correlational strength of shared features only. Taxonomic features (e.g., *is an animal*), which arguably are not features at all, were not included in the calculation of mean distinctiveness, mean correlational strength, or NOF (McRae et al., 2005; Taylor et al., 2012). Mean correlational strength was log‐transformed to give a more normally distributed predictor variable.^1^

Our experiment used a correlational rather than a factorial design, where the effects of the theoretically less interesting variables could also be included in the analysis along with the theoretically interesting predictors of mean distinctiveness and mean correlational strength. This approach has the advantage of using a much larger set of concept words than is possible with factorial designs. Generating sets of well‐matched stimuli for factorial designs may result in unrepresentative sets of experimental items, since factors that are orthogonal with respect to the items in the experiment are often not actually orthogonal in the entire population of items from which the stimuli are sampled, leading to problems of generalizability (Baayen, 2010; Balota, Cortese, Sergent‐Marshall, Spieler, & Yap, 2004; Hogarth, 2005). For our predictions regarding the effects of feature statistics at early and late stages of processing, we again chose a correlational approach over a factorial approach. Rather than attempting to manipulate the time course of participants’ responses through experimenter‐chosen timing parameters (e.g., different response deadline conditions, which are difficult to achieve satisfactorily with spoken stimuli), we instead analyze how effects of feature statistics vary as a function of participants’ overall response speed. In this way, we avoid sampling a relatively small set of possible timing manipulations, which may give an incomplete picture of the dynamics of conceptual processing (see Mirman & Magnuson, 2009, for a similar argument against timing manipulations in priming paradigms).

#### 2.1.3 Procedure

Participants were tested in small groups in a sound‐attenuated room. They listened to the stimuli over headphones and were instructed to press a button labeled “yes” with their dominant hand when the stimulus was a real word in English and a button labeled “no” with their other hand when it was not a real word. They were asked to do the task as quickly and as accurately as possible. There were four blocks of trials, with words and non‐words evenly distributed over blocks. The order of items was pseudo‐randomized within each block, and the order of the four blocks was randomized across subjects to ensure that there were no systematic practice or fatigue effects for items. Response times were measured from stimulus onset, and the timeout was 2,500 ms. The inter‐trial interval was 1,200 ms. The experiment lasted approximately 45 min.

#### 2.1.4 Statistical analyses

Since high collinearity between predictor variables results in unstable estimated coefficients (Baayen et al., 2006), we assessed collinearity between the (mean‐centered) variables by calculating the condition index (Belsley, Kuh, & Welsch, 1980). The condition index was 2.69, indicating low and acceptable collinearity between our stimulus variables (Belsley et al., 1980). Details of the descriptive characteristics of the experimental items and a table of the correlations between the predictors are provided in the Supplementary Material (Tables S1 & S2).

Response time and error data were analyzed with mixed effects models with by‐subject and by‐item random intercepts in R (R Development Core Team, 2008) with the lme4 (Pinheiro & Bates, 2000), languageR (Baayen, 2008), and LMERConvenienceFunctions (Tremblay & Ransijn, 2012) libraries. Four session‐specific variables relating to other effects such as fatigue were also included in the mixed models: (a) *trial order* (the location of each item in the presentation sequence), (b) whether the subject had responded with the same hand on the preceding trial (*previous response same*), (c) whether the participant had made an error on the previous trial (*previous response error*), and (d) the *RT on the previous trial* (the inverse RT of the preceding trial with the inverse duration of that stimulus partialed out). Model fitting was done following the iterative procedure outlined by Tremblay and Ransijn (2012). We began with a full model, including all session and predictor variables as fixed effects and random intercepts for subjects and items. Non‐significant session variables were then iteratively removed from the full model by a backwards elimination procedure until only significant variables remained. We then iteratively forward‐fit random effects to include all random slope parameters that gave a significant improvement in model fit. Given our hypotheses concerning mean distinctiveness and mean correlational strength, fixed effects for these variables were forced into the final model irrespective of their significance. We checked for nonlinear relationships between inverse RT and the mean distinctiveness and mean correlational strength variables using restricted cubic spline functions with three knots, at quantiles 0.1, 0.5, and 0.9 (the default quantiles of the rcs function in the R rms package). No nonlinear effects were found (¦*t*¦ < 1). We also tested for an interaction of the mean distinctiveness and mean correlational strength measures, which was not significant (¦*t*¦ < 1). Nonlinear and interaction terms were therefore not included in the final model. The *p*‐values for the RT models are derived from 1,000,000 samples from the posterior distribution of the parameters of the fitted model using Markov Chain Monte Carlo methods (Baayen et al., 2008). We first present the results of a standard mixed model analysis that includes all participants. Given our hypothesis that conceptual processing involves general‐to‐specific activation mediated by early effects of feature sharedness and later effects of correlational strength within the concept, we also investigate how conceptual structure effects vary as a function of participants’ overall response speed.

### 2.2 Results

The mean and standard deviation of correct real‐word responses across all items and participants were 853 and 238 ms, respectively. RT data were inverse‐transformed (Ulrich & Miller, 1994) and the harmonic mean was 803 ms. The fixed effects from the mixed effects analyses are presented in Table 1. The model fitting procedure (see “2.1.4”) resulted in by‐subject random slope parameters being added for duration, phoneme rate, lemma frequency, familiarity, and speed on previous trial. The results show predictable effects for the session and control variables. Participants’ lexical decision latencies were faster when they had been fast on the preceding trial, and when the previous response had been an error. Response times were faster for more frequent words, more familiar words, and words with higher phoneme rate. RTs were slower for longer words. RTs were facilitated by a greater number of semantic features, consistent with earlier NOF findings in lexical decision in the visual modality (Grondin et al., 2009; Pexman et al., 2002). We found a significant facilitatory effect of feature sharedness across concepts (i.e., an inhibitory effect of the *mean distinctiveness* measure), indicating that participants are faster to respond to concepts with relatively more shared features, consistent with our predictions. There was no significant effect for *mean correlational strength* within the concept, although there was a facilitatory trend.

**Table 1:** Fixed effects from the mixed effects model of the inverse‐transformed lexical decision RT data

	Estimate	*SE*	*t*‐value	*p*‐value
Previous response error	0.018	0.009	1.994	.046
Speed on previous trial	0.052	0.004	12.547	<.001
Duration	<0.001	<0.001	−9.380	<.001
Phoneme rate	0.015	0.003	5.555	<.001
Lemma frequency	0.011	0.004	2.522	.006
No. of features	0.005	0.002	2.967	.001
Familiarity	<0.001	<0.001	2.376	.010
Mean distinctiveness	−0.072	0.032	−2.263	.013
Mean correlational strength	0.022	0.017	1.324	.146

The error rate on real‐word trials was 5.2%. Error data were analyzed with a generalized mixed effects model (Table 2). Random slope parameters for familiarity were included in the final model. As in the RT analysis, significant effects followed predicted patterns. Participants made fewer errors on more familiar words, on longer words, on words with higher phoneme rate, and when they had made an error on the preceding trial. Participants made more errors when they responded using the same hand on the previous trial, when they were near the end of the testing session (Trial Order), and when they were fast on the preceding trial. There were no effects of mean distinctiveness or mean correlational strength in the error analysis.

**Table 2:** Fixed effects from the mixed effects model of the lexical decision error data

	Estimate	*SE*	*t*‐value	*p*‐value
Previous response same	1.201	0.097	12.365	<.001
Previous response error	−0.655	0.209	−3.128	.002
Speed on previous trial	0.139	0.050	2.794	.005
Trial order	0.162	0.042	3.890	<.001
Duration	−0.281	0.076	−3.695	<.001
Phoneme rate	−0.357	0.076	−4.718	<.001
Familiarity	−0.446	0.091	−4.927	<.001
Mean distinctiveness	0.094	0.078	1.200	.230
Mean correlational strength	−0.066	0.074	−0.887	.375

**Figure 2:**
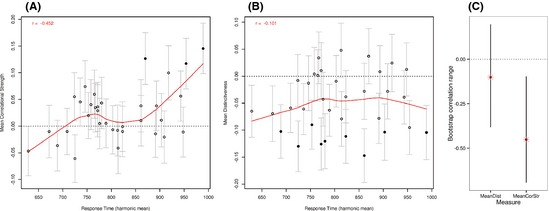
Participants’ sensitivity to (A) mean correlational strength (i.e., the standardized beta‐estimate for correlational strength from the regression model fitted for each participant) and (B) mean distinctiveness, as a function of their mean RT. The results show a greater facilitatory effect of correlational strength for slower participants, but no difference in the effect of mean distinctivess as a function of participant mean RT. Filled circles indicate effects that were significant in that individual participant's regression, open circles indicate effects that were not significant in that individual's regression. Error bars for each participant represent that participant's standard error for the beta estimate. The red line depicts the locally weighted scatter plot smoothing (LOWESS) curve. (C) 95% confidence intervals for the mean correlational strength and mean distinctiveness correlations reported in (A) and (B), based on bootstrap resampling of participants (see “Results”). Red circles are the mean values of the correlation statistics across bootstrap samples. Red asterisks (overlapping with the circles) are the correlation values for the 34 subjects reported in (A) and (B).

Our main predictions concerned the influence of feature statistics across the time course of spoken word processing. Using a correlational approach, we tested these predictions by investigating how the influence of distinctiveness/sharedness and correlational strength varied as a function of participants’ overall speed. We conducted a multiple regression analysis for each individual participant with the same set of predictor variables as used in the mixed model of RT (following the method of random regression; Lorch & Myers, 1990). These models generated standardized beta estimates of the influence of each variable for each subject, which we then correlated with participants’ mean inverse‐transformed RTs, to test whether the effect of each variable varied as a function of overall response speed. First, we focus on the two theoretically interesting variables, mean distinctiveness and mean correlational strength. Consistent with our prediction that slower participants should be more sensitive to the dynamics of network settling, participants with slower mean response latencies showed a greater facilitatory effect of mean correlational strength than faster participants (the effect of mean correlational strength correlated negatively with participants’ mean inverse‐transformed RT; *r *=* *−.45, *p *=* *.007; see Fig. 2A). The corresponding analyses with the mean distinctiveness variable showed that the influence of distinctiveness/sharedness did not vary with speed of processing (*r *=* *−.10, *p *=* *.56; Fig. 2B). In order to check whether the significant correlation for the mean correlational strength effect depends on this particular sample of subjects, we conducted a bootstrap resampling of the correlation coefficients to obtain a bootstrap confidence interval and bootstrap *p*‐value for the correlation coefficient test statistic (see, e.g., Efron & Tibshirani, 1986; bootstrap computed using the R boot package). We took 10,000 samples of 34 subjects, drawn with replacement from the set of 34 subjects, creating a distribution of correlation coefficients. Consistent with the parametric *p*‐value reported above, the 95% confidence interval did not intersect zero (bias‐corrected 95% CI: lower bound = −0.69; upper‐bound = −0.11; Fig. 2C). The bootstrap analysis therefore supports the conclusion that our significant mean correlational strength correlation is robust with respect to the sampling distribution of the correlation statistic.

Is the correlation for the correlational strength effect greater than the correlation for the distinctiveness effect? To answer this question, we used a permutation test. We built up a null distribution for the difference between the distinctiveness and correlational strength correlations by permuting subject mean RTs 10,000 times, and calculating the difference in Pearson correlation values for distinctiveness and correlational strength for each permutation. The one‐sided permutation based *p*‐value for the difference in the correlation values was 0.048, indicating that the relationship between the correlational strength effect and mean RT is significantly stronger than that between distinctiveness and mean RT.

For the other predictor variables included in the mixed effects model, only the influence of duration showed a significant relationship with mean RT (*r *=* *.69, *p *<* *.001; see Fig. S1A); this is consistent with the hypothesis that faster participants’ lexical decisions are more strongly influenced by surface properties of the word form whilst those of slower participants are relatively more influenced by later semantic settling. None of the other session or concept predictors showed significant correlations (*p*'s > 0.1; Fig. S1).

The pattern of results for the mean distinctiveness and mean correlational strength measures suggests that while both fast and slow participants are sensitive to the early activation of shared features, slow participants are further influenced by the gradual emergence of a coherent core semantic representation driven by strong correlation between features within the concept. This interpretation assumes that slow responders differ from fast responders in that they have a higher threshold of semantic evidence for making their response. Although we cannot discount the possibility that fast and slow participants may have the same response threshold but differ in speed for other reasons—some participants may simply be cognitively faster than others—this possibility does not account for the difference in the effect of mean correlational strength for fast and slow responders relative to other predictor variables. If it were the case that slow responders respond on the same basis as the fast responders, except more slowly, then there should not be differences in the effects due to conceptual structure.

## 3 Experiment 2: Simulation study

We subsequently tested our predictions in a connectionist simulation that modeled the activation of meaningful concept features. Recurrent connectionist simulations enable us to describe the flow of activation through interconnected semantic feature nodes over time, and they allow us to measure the influence of feature statistics on the evolution of meaning.

### 3.1 Method

#### 3.1.1 Network architecture

The present simulations were based on the two‐layer attractor network model of Cree et al. (2006), with a 30‐node input layer connected to a 2,341‐node recurrently connected semantic layer (a node for each feature in the anglicized McRae norms). However, the network of Cree et al. (2006) simulates a word reading task, mapping orthography to semantics, and as such all word form information is available immediately to the model: From the first time‐tick, corresponding to stimulus onset, an activation pattern representing the written word is presented on the input layer of the network. The target semantic pattern emerges on the semantic feature layer gradually, over a series of processing time‐ticks.

In order to adapt this architecture to spoken word comprehension, in our implementation the input is presented to the model gradually, with a new bit of information (i.e., input node) provided to the model on each time‐tick. On the first time‐tick, only the first node of the 30‐node input layer is provided (i.e., clamped to its input value), on the second time‐tick, the first and second nodes are provided, and so on, until all 30 input nodes making up the full input pattern are provided at time‐tick 30 (time‐tick 30 therefore corresponds to the offset of the auditorily presented word form). Activation in the semantic layer continues to unfold for an additional 20 time‐ticks, for a total of 50 processing time‐ticks (in the original Cree et al. model, activation unfolds over 20 time‐ticks after word form presentation). Whereas the input in the Cree et al. simulations represented orthography, here it represents phonology, and the temporally sequential nature of the phonological input is explicitly modeled. The model was implemented using the MikeNet V8.02 package (http://www.cnbc.cmu.edu/~mharm/research/tools/mikenet/).

As in Cree et al. (2006), the input patterns are abstract representations of the word forms, with each word corresponding to a unique random pattern consisting of an activation value of 1 on 3 of the 30 input nodes and an activation value of 0 on the other 27 nodes. This gives input patterns which are highly overlapping (representing overlapping phonology in our case) and which model the arbitrary nature of the mapping from word form to semantics. In particular, at early time‐ticks, the partially presented input pattern for a particular item will be consistent with a cohort of several word forms, and this cohort diminishes as more input becomes available until a uniqueness point is reached and only one word remains consistent with the input.

#### 3.1.2 Training

The simulation was trained using all 517 concepts in the anglicized McRae norms. The network was trained using continuous recurrent back‐propagation through time (Plaut, McClelland, Seidenberg, & Patterson, 1996). During training, cross‐entropy error (i.e., the measure of agreement between the target and observed activation patterns) was evaluated over all 50 time‐ticks (error is evaluated over the final 10 time‐ticks in the original Cree et al. model). This was done so that the model would be trained to try and activate semantic features consistent with the target concept as soon as possible, before the offset of the word form and before the word form pattern has become consistent with a single specific concept. This design decision implements a principle of interactive models of spoken word recognition such as the cohort model, where semantic information is activated as rapidly as possible, even before a word's recognition point (Gaskell & Marslen‐Wilson, 2002; Marslen‐Wilson, 1987; Moss et al., 1997).

In the trained model, activation propagates from the input layer to the semantic layer, eventually producing the correct activation pattern on the semantic layer. The learned weights between input and semantic layers capture a mapping of activation between pairs of nodes on the two layers and will be different depending on the particular values on the input nodes. Given that the input representations are arbitrary patterns, we conducted separate simulations with 30 different independent sets of randomly generated input patterns, to ensure that our results properly generalized over the set of possible word form‐to‐semantics mappings.

In recurrent back‐propagation networks, a node *n*'s input at time *t*,* x*_*n*_(*t*), is calculated as a weighted average of its actual external input at that time‐tick, *e*_*n*_(*t*) (i.e., the summation of incoming activation from other connected nodes), and its input at the previous time‐tick, *x*_*n*_(*t*−1) (Plaut et al., 1996; see also O'Connor, Cree, & McRae, 2009):

$$x_{n}\left(t\right)=ce_{n}\left(t\right)+\left(1-c\right)x_{n}\left(t-1\right)$$

Holding the total number of time‐ticks in the simulation constant, the free parameter *c* controls the speed at which a node responds to changes in its external input (when *c* is close to 1, activation is mostly influenced by the current external input and can change quickly; when *c* is close to 0, activation is mostly influenced by the input on the previous time‐tick and changes slowly). In Cree et al.'s simulations, *c* is chosen as 0.2 (following Plaut et al., 1996, and the MikeNet default settings). However, in our case, the choice of *c* reflects a particular relationship between the rate of phonological change in the input (1 node per time‐tick) and the rate of activation change on the semantic layer. We therefore ensured that our results generalized over different values of *c* (0.16, 0.2, & 0.24).

In total therefore there were 90 separate trained models (30 sets of input patterns and 3 choices of *c*), which furthermore were initialized with different activation values and connection weights (random values in the range 0–0.1 for initial activations and in the range 0–0.05 for initial connection weights). Other training details were the same as Cree et al. (2006).

#### 3.1.3 Evaluation and statistical analyses

To simulate the activation of meaning from spoken word input, we presented the word form pattern associated with each concept to the trained models on the input layer (gradually over 30 time‐ticks, as in training) and recorded the activation levels of the target features of that concept on the output layer for each of the 50 time‐ticks. For each of the 90 trained models, we conducted five evaluation runs, again with different randomizations of the initial activation values, and averaged the resultant activations over the five runs.

We analyzed the simulation output in two ways. Since the simulation output records the activation values of each individual feature node, we first analyzed the output in terms of individual features. Secondly, we analyzed the model output at the level of concepts, averaging the target feature activations within each concept. For this analysis, we determined how mean concept activation was influenced by the concept‐level measures of mean distinctiveness and mean correlational strength (the same variables we used in the behavioral analyses) at each time‐tick. This analysis is analogous to the by‐subject regression analysis of the behavioral RT data, with a separate regression model fit at each time‐tick to evaluate the influence of the two measures at each time‐tick.

### 3.2 Results

#### 3.2.1 Early activation of shared features

**Figure 3:**
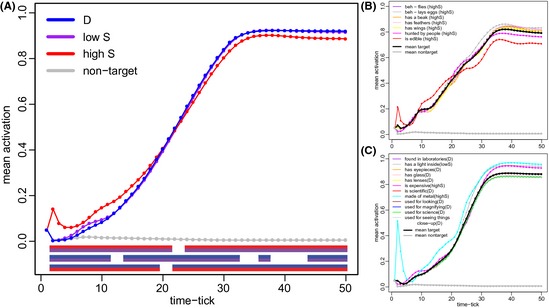
The simulated time course of activation for shared (S) and distinguishing (D) features. (A) The mean activation patterns for the shared and distinguishing features of target concepts. The shared features are further divided by a median split into those that are moderately shared (occurring in 3–17 concepts; low S) and those that are highly shared (occurring in 18 or more concepts; high S). The number of D, low S, and high S feature instances are approximately equal. Shared features, and in particular high S features, have higher initial activation. *Non‐target* features are features outside of the target concept, which should not be activated by the model. The three bands underneath the plot depict the time intervals when there is a significant difference (*p* < .05, Bonferroni corrected for number of time‐ticks and number of contrasts) between the three pairs of feature types (first band: “high S” vs. “low S”; second band: “D” vs. “low S”; third band: “high S” vs. “D”; the pair of colors in each band represents the pair of groups being compared). (B) The time course of feature activation over the 50 time‐ticks for the low mean distinctiveness concept *pheasant* (all features are high S). Averaging across features, *pheasant* has relatively high initial activation. (C) The time course of feature activation for the high mean distinctiveness concept *microscope*. Averaging across features, microscope has relatively low initial activation.

The average activation values for shared and distinguishing features, averaging over the 90 training instances and over all feature instances in all concepts, are shown in Fig. 3A. Because approximately 2/3 of all features are shared (4,273 shared vs. 2,050 distinguishing), we further divided the shared features into highly shared (high S) and moderately shared (low S) groups, based on a median split of their distinctiveness values. The numbers of distinguishing (D), low S and high S feature instances in the model are therefore comparable (2,050, 2,129, and 2,144, respectively). Consistent with the results described by Cree et al. (2006; Simulation 1), distinguishing features were more strongly activated than shared features at most time‐ticks (ticks 22–50; Fig. 3A). However, at early time‐ticks (2–21), activation for shared features, and in particular the high S features, was significantly greater than for distinguishing features.

Although shared features within the target concept have high activation even as early as the second time‐tick, early activation patterns are necessarily diffuse and include non‐target features (see Fig. 1). This is because the word form input exerts a stronger influence on activation at early time‐ticks, and features not in the target concept but consistent with the presented word form pattern will also tend to be activated. Our word form patterns were generated randomly (following Cree et al.) and so, on average, features occurring in many concepts will be consistent with more input patterns. For example, *made of metal* and *is large* are strongly activated for *pheasant* at early time‐ticks, because many of the concepts that have word form patterns overlapping with the word form pattern for *pheasant* also share these features. Thus, the simulation is consistent with an account in which semantic information about phonological cohort competitors initially becomes activated (see “4”). In the simulation, the diffuse activation driven by the input gives way to more specific semantic representations driven by feature correlation. Similar results are found irrespective of the choice of the speed parameter or the choice of word form patterns.

The temporal pattern of activation of shared and distinguishing features in the connectionist model is thus consistent with our general‐to‐specific hypothesis for the activation of meaning. More specifically, on the assumption that the early activation of general shared information is sufficient evidence for making lexicality judgments, the attractor network data support an interpretation of our behavioral findings in which concepts with many highly shared features (such as *pheasant*; Fig. 3B) are facilitated in lexical decision compared to concepts with more distinctive features (e.g., *microscope*; Fig. 3C) because the former induce more early, shared‐feature semantic activation that facilitates a rapid response.

#### 3.2.2 Concept activation and mean distinctiveness

**Figure 4:**
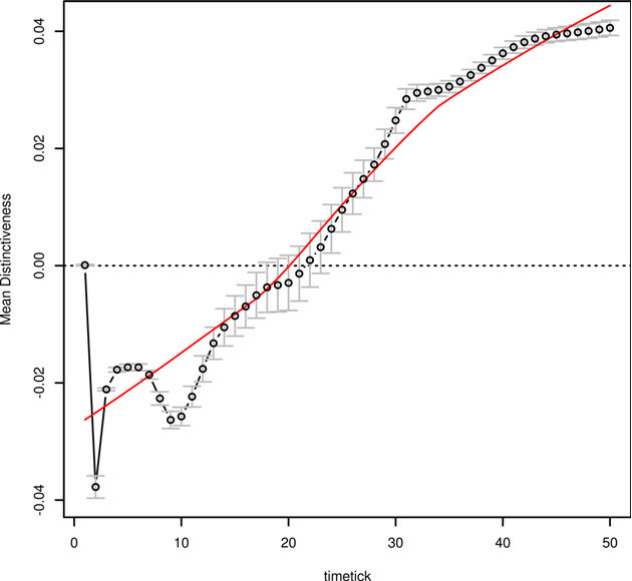
The influence of mean distinctiveness on the mean activation of target concepts in the simulation (i.e., the standardized beta‐estimate for mean distinctiveness from the regression model fitted at each time‐tick), presented as a function of time‐tick. The plot shows an initially facilitatory effect of sharedness (negative beta‐estimates), with a later facilitatory effect of distinctiveness (positive beta‐estimates).

The behavioral experiment measured RTs to concepts rather than to individual features, and the variables used in the corresponding analyses represented summary measures of whole concepts (i.e., mean distinctiveness of the concepts’ features and mean correlational strength of the concepts’ features). Our second analysis of the simulation therefore examined how the mean activation of concepts’ features at each time‐tick related to the mean distinctiveness and mean correlational strength measures used in Experiment 1. In the same manner as the linear regression models were fitted for each subject individually in Experiment 1, we fitted linear regression models for each time‐tick of the model's time course. As a proxy for RT, we used mean activation strength of target features as the dependant variable (Cree et al., 2006; see also Laszlo & Plaut, 2012). The theoretically important predictor variables were the same as in Experiment 1 (the lexical, phonological and other variables used in Experiment 1 were not included as the model is naïve to these factors). Fig. 4 presents the beta estimates reflecting the influence of mean distinctiveness at each time‐tick. Consistent with the feature‐based analysis above, there is a facilitatory effect of sharedness at early time‐ticks (negative beta‐estimates for mean distinctiveness up to tick 21). At later time‐ticks, concepts with more distinctive features are facilitated. This pattern of results is consistent with the hypothesis that conceptual activation becomes increasingly specific over the time course of processing. We note that a facilitatory effect of distinctiveness was not observed for the slower participants in the behavioral study, consistent with our claim that this information is not required for the task. Instead, slow participants appear to be influenced by shared, correlated features giving category‐level evidence (e.g., “large cat”; Fig. 2B) rather than distinctive features.

#### 3.2.3 Concept activation and mean correlational strength

**Figure 5:**
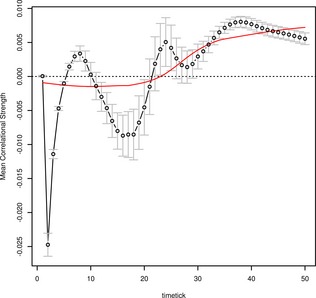
The influence of mean correlational strength on the mean activation of target concepts in the simulation (i.e., the standardized beta‐estimate for mean correlational strength from the regression model fitted at each time‐tick), presented as a function of time‐tick. The plot shows a late facilitatory effect of correlational strength.

Fig. 5 presents the beta estimates for the influence of mean correlational strength at each time‐tick. The effect of correlational strength is weaker than distinctiveness in general and is highly variable at early time‐ticks (and highly sensitive to the particular word form patterns used, as indicated by the large error bars). However, after the offset of the word form input (time‐tick 30) mean correlational strength settles into a clear facilitatory pattern. Taken together, these results are in line with our interpretation of the different feature statistics effects for fast and slow responders—fast responders respond on the basis of early shared feature activation, whereas slow responders respond on the basis of later and more specific activation, which is driven by strong correlations between features within the concept.

## 4 Discussion

The present results provide new evidence on how conceptual structure statistics affect spoken word processing. Across all participants, lexical decisions were facilitated when concepts had many shared features, whereas only slow participants also showed facilitation for concepts with greater correlational strength between their shared features. The computational simulations confirmed these effects of feature statistics, with stronger activation of shared features at early time‐ticks and a positive association between mean correlational strength and concept activation at later time‐ticks.

According to distributed models of conceptual representations such as the CSA (Tyler & Moss, 2001b; Tyler, Moss, et al., 2000) and McRae and colleagues’ model (Cree et al., 2006; McRae et al., 1997), the activation of a feature within a distributed semantic system is a function of its correlational strength with other features, with highly correlated features activated faster than weakly correlated features. That the fast participants are not sensitive to the concepts’ mean correlational strength suggests that they make their decisions before activation has begun to settle into a network of core intercorrelated features, giving relatively less weight to semantic evidence when making their judgements. We argue that the lexical decision performance of slow participants was influenced by the speed with which a coherent set of interconnected features within the concept activated. This account is consistent with Cree et al. (1999), whose simulations examined the relationship between overall concept settling rate and concept intercorrelational density, and showed the strongest influence of intercorrelational strength at later time‐ticks (Cree et al., 1999; Table 1). Within a concept, the effect of mean feature correlation is most pronounced at later time‐ticks, when the concept representation is settling.

In the case of feature distinctiveness/sharedness, the results from feature verification experiments have been varied. Cree et al. (2006) reported a facilitatory effect of feature distinctiveness, with distinctive features verified faster than shared features. This contrasts with the facilitatory effects of sharedness in the present lexical decision study, and also with the feature verification results of Randall et al. (2004), who found an inhibitory effect of feature distinctiveness for living‐thing concepts. Facilitatory effects of sharedness are also in line with evidence showing that high numbers of shared features are facilitatory in lexical and semantic decision to written words (Grondin et al., 2009). The apparent discrepancy here can be resolved by a more detailed account of the dynamics of processing, where the influence of sharedness/distinctiveness on processing can vary over time and with task demands. Cree et al.'s task paradigm used relatively slow timing parameters, and therefore may have been more suited to tapping later and higher‐order stages of conceptual processing compared with the “beat the beep” paradigm used by Randall et al. (see Taylor et al., 2008, for a discussion). Furthermore, effects of shared and distinctive features may interact with the information demands of a particular task, with distinctive features exerting less influence when specific concept information is not required (e.g., domain or lexical decision; Bright et al., 2007; Grondin et al., 2009; Taylor et al., 2012).

Similar arguments can be made for the interpretation of the effects of distinctiveness and correlational strength in the present computational model. We chose the Cree et al. (2006) architecture as the starting point for our simulations because it implements a distributed feature‐based semantic system and because it aims to simulate the dynamics of conceptual processing unfolding over time. Cree et al. showed that their simulation accounted for the primacy of distinctive features effect that they observed in their behavioral results and, indeed, in our simulations based on this model we also observe stronger activation of distinctive features at later time‐ticks. This is not surprising given that the model was trained to activate specific target concept representations. However, Cree's architecture used instantaneous input representing word orthography. When we adapted the architecture to make it more compatible with continuous speech and consistent with the principles of the cohort model (Marslen‐Wilson, 1987), the relationship between feature distinctiveness/sharedness and activation was not constant over time, with shared features in fact showing stronger activation at earlier time‐ticks. This shows that, with appropriate modifications, a distributed feature‐based attractor network model of processing dynamics can account for both the current spoken word lexical decision data, as well as the Cree et al. feature verification results.

Our interpretation of the early sharedness effects and later correlational strength effects in the model's dynamics is also supported by Mirman and Magnuson's simulations with the Cree et al. (2006) network architecture (Mirman & Magnuson, 2009). They examined the activation of target concepts over time, together with semantically near and distant neighbors of those targets (semantic distance was measured as cosine similarity). Consistent with our claims about general‐to‐specific activation, they found that activation of semantically distant neighbors was highest at early time‐ticks and gradually decreased as processing progressed. Activation of semantically near neighbors peaked later, before declining as activation settled on the specific target concept. This later peak for concepts semantically close to the target is consistent with the activation of a coherent network of shared, correlated features within the target representation (e.g., the features that compose *large cat* if the target concept is *tiger*, which will also be features of the near neighbors of tiger) and corresponds to the level of processing at which we claim the slower participants make their responses.

General‐to‐specific dynamics in conceptual activation are also compatible with the results from recent studies of object processing. Shared features facilitate object processing tasks which require a general conceptual representation (e.g., domain decisions; Taylor et al., 2012), whereas distinctive features are facilitatory when the object must be uniquely identified. In an MEG object naming study (Clarke, Taylor, Devereux, Randall, & Tyler, 2010; Clarke et al., 2011) we found initial perceptual effects of visual object stimuli in posterior sensory regions (occipital cortex at 70–120 ms) followed by an effect (at 80–220 ms) of an increasing number of shared features reflecting coarse, category‐level information in more anterior regions along the ventral stream. At later latencies (220–280 ms), there were continued effects of sharedness and additionally an effect of the correlational strength of shared features (as well as effects of feature distinctiveness and the correlational strength of more distinctive features, as predicted by the CSA for tasks which involve basic‐level identification). These findings are consistent with our claim that shared features yield early, general semantic activation, whereas the effect of correlational strength occurs later and determines the speed with which activation becomes focussed on a specific region of semantic space.

Our findings also reflect the general‐to‐specific nature of lexical processing proposed in the cohort model (Marslen‐Wilson, 1987; Marslen‐Wilson & Welsh, 1978). In the cohort model, the incoming acoustic signal initially activates a large word‐initial cohort of lexical competitors that match the input. This set of competitors shrinks as members of the cohort become inconsistent with the incoming signal. At the point in the auditory sequence when only one word remains that is consistent with the input the word is recognized (the “recognition” point). In terms of a distributed model of word meaning, this account proposes that early in the time course of word recognition, activation is spread over a broad area of semantic space (a “semantic blend,” corresponding to initial activation of the meanings of the competitors in the cohort), which progresses gradually toward the specific target representation as competitors decay from the cohort (Gaskell & Marslen‐Wilson, 1997; Marslen‐Wilson, 1987). For example, highly shared features like *made of metal* may have some early initial activation for *pheasant*, because many of the competitors in the initial cohort of *pheasant* will have this feature (e.g., *fence*,* fender*,* ferry*). Combining the cohort model with a feature‐based statistical approach to conceptual semantics, we speculate that the correlational strength of features in the target concept may be one variable influencing the dynamics of how competitors decay from the cohort over time; that is, the speed with which the early, general semantic activation of cohort competitors decays is proportional to how quickly the target shared‐correlated feature network becomes activated.

Although we have focused on the role of semantic feature statistics in our analysis of the lexical decision data, it is important to note that semantic activation is only one of several components influencing participants’ lexical decisions, and we have not attempted to explicitly model the effects of these additional factors on the lexical decision process. For example, for a hypothetical non‐word such as *pheasanp*, semantic features will initially activate as for *pheasant*, providing semantic evidence for lexicality that will presumably interfere with the ability to make a correct non‐word judgement, resulting in more errors and longer reaction times. Lexical decision likely involves the complex integration of different kinds of phonological and semantic evidence and their associated decision thresholds (which is outside the scope of the current study, given its focus on conceptual structure statistics). Clearly, it will be critical for future research to integrate different models of the factors influencing lexical decision.

It is also important to note that feature‐based statistical approaches to concepts are not intended to capture the full richness of conceptual knowledge. Instead, they account for a specific aspect of conceptual knowledge—the network topology of the distributed conceptual system. In particular, statistical measures do not explicitly represent the conceptual domain of features (e.g., whether a feature is a feature of living things or of non‐living things) or the features’ sensory modalities (e.g., whether a feature reflects a visual property or a motor‐function property). Nor do the conceptual structure measures capture aspects of meaning that cannot easily be accounted for within a purely feature‐based framework, such as how concepts and their interrelationships are embedded in richer pragmatic knowledge about the world (Komatsu, 1992; Murphy & Medin, 1985). Rather than capturing the entirety of conceptual *content*, feature‐based statistics are primarily focused on reflecting conceptual *structure,* allowing us to test specific claims about how the internal structure of concepts and the relationship between concepts influence the processing of meaning.

In summary, our experiments investigated the role of feature statistics in spoken word recognition. We found that statistical properties of concepts’ features—their distinctiveness/sharedness and correlational strength—significantly influence the processing of spoken words. This is important as it demonstrates that the mapping of form onto meaning in spoken language comprehension depends on the structural properties of the features of the target concept.

## Supporting information


**Table S1.** Descriptive characteristics (means and standard deviations, SD) of the experimental items. Data for lemma frequency and mean correlational strength are in retransformed units.
**Table S2.** Pearson correlation values for pairs of concept predictor variables included in linear effects model.
**Fig. S1.** Participants’ sensitivity to the less theoretically relevant concept variables as a function of their mean RT. Filled circles indicate effects that were significant in that individual participant's regression; open circles indicate effects that were not significant in that individual regression. Error bars for each participant represent that participant's standard error for the beta estimate. The red line depicts the locally weighted scatter plot smoothing (LOWESS) curve. (a) Word duration, *r *= −.692, *p* < .001; (b) familiarity, *r* = .243, *p* = .17; (c) number of features (NOF), *r* = −.061, *p* = .73; (d) lemma frequency, *r* = .240, *p* = .17; (e) phoneme rate, *r* = .162, *p* = .36.

## Note

1 Predictor variables were transformed when their distribution was non‐normal (absolute value of skewness and/or kurtosis greater than 3) and transforming gave a more normal distribution. For RTs, raw, inverse, and log transformed data were visually inspected and statistically tested for normality, and the inverse transform was clearly the most appropriate for these data. Results remain the same if raw variables are used in the reported behavioral analyses.
