# Supplementary material for: Feature Statistics Modulate the Activation of Meaning During Spoken Word Processing
Source: Cogn Sci. 2015 Jun 4;40(2):325–50. doi: 10.1111/cogs.12234 (PMC4949631; doi:10.1111/cogs.12234)
Supplement: Supplementary file 1 — Table S1. Descriptive characteristics (means and standard deviations, SD) of the experimental items. Data for lemma frequency and mean correlational strength are in retransformed units. Table S2. Pearson correlation values for pairs of concept predictor variables included in linear effects model. Fig. S1. Participants’ sensitivity to the less theoretically relevant concept variables as a function of their mean RT. Filled circles indicate effects that were significant in that individual participant's regression; open circles indicate effects that were not significant in that individual regression. Error bars for each participant represent that participant's standard error for the beta estimate. The red line depicts the locally weighted scatter plot smoothing (LOWESS) curve. (a) Word duration, r = −.692, p < .001; (b) familiarity, r = .243, p = .17; (c) number of features (NOF), r = −.061, p = .73; (d) lemma frequency, r = .240, p = .17; (e) phoneme rate, r = .162, p = .36. [file COGS-40-325-s001.docx]

**Supplementary Material**

Table S1. Descriptive characteristics (means and standard deviations, SD) of the experimental items. Data for lemma frequency and mean correlational strength are in retransformed units.

|  | Duration | Phoneme rate | Lemma frequency | Nr. of features | Mean Distinctiveness | Mean Corr. Strength |
| --- | --- | --- | --- | --- | --- | --- |
| Transform | none | none | log | none | None | log |
| Mean | 591 | 8.28 | 122 | 12.3 | 0.328 | 0.256 |
| SD | 124 | 1.98 | 25, 578* | 3.3 | 0.164 | 0.18,0.34* |

** 1 standard deviation above and below the mean in retransformed units*

Table S2. Pearson correlation values for pairs of concept predictor variables included in linear effects model fitting.

|  | Mean Distinctiveness | | Duration | | Phoneme rate | | Lemma frequency | Mean Corr. Strength | Familiarity |
| --- | --- | --- | --- | --- | --- | --- | --- | --- | --- |
| Nr. of features | 0.13 | | -0.03 | | -0.06 | | 0.20 | 0.02 | 0.32 |
| Mean Distinctiveness |  | | 0.01 | | -0.03 | | 0.23 | -0.23 | 0.30 |
| Duration |  | |  | | 0.17 | | -0.37 | 0.05 | -0.19 |
| Phoneme rate |  |  | |  | | -0.34 | | 0.03 | -0.27 |
| Lemma frequency |  |  | |  | |  | | -0.05 | 0.65 |
| Mean Corr. Strength |  |  | |  | |  | |  | -0.09 |


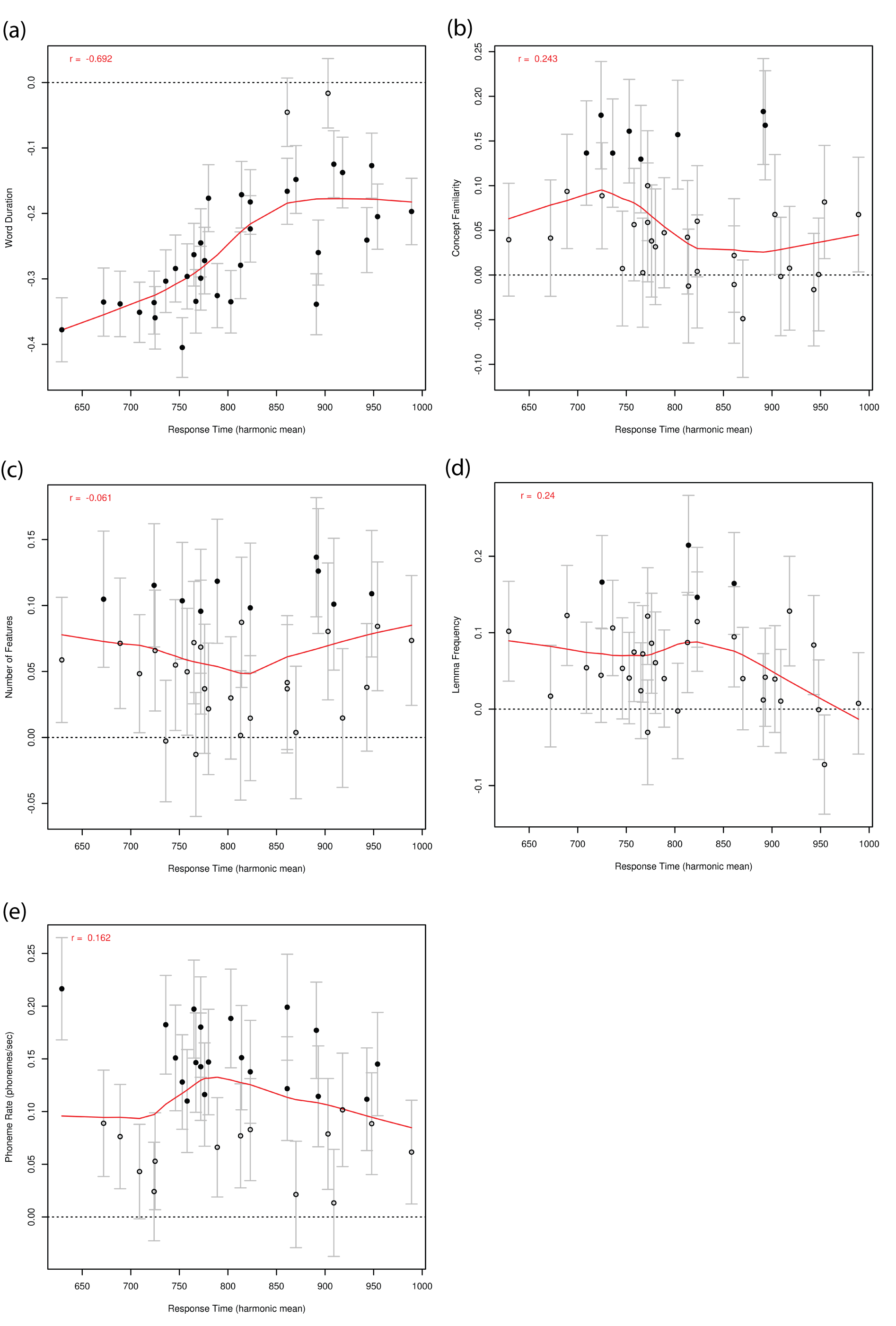


Figure S1. Participants’ sensitivity to the less theoretically relevant concept variables as a function of their mean RT. Filled circles indicate effects that were significant in that individual participant’s regression, open circles indicate effects that were not significant in that individual subjects regression. Error bars for each participant represent that participant’s standard error for the beta estimate. The red line depicts the locally-weighted scatterplot smoothing (LOWESS) curve. (a) word duration, *r* = -0.692, *p* < 0.001; (b) familiarity, *r* = 0.243, *p* = 0.17; (c) number of features (NOF), *r* = - 0.061, *p* = 0.73; (d) lemma frequency, *r* = 0.240, *p* = 0.17; (e) phoneme rate, *r* = 0.162, *p* = 0.36.
